# Experimental In Vivo Models of Candidiasis

**DOI:** 10.3390/jof4010021

**Published:** 2018-02-06

**Authors:** Esther Segal, Michael Frenkel

**Affiliations:** Department of Clinical Microbiology and Immunology, Sackler School of Medicine, Tel-Aviv University, Tel-Aviv 69978, Israel; mifren10@gmail.com

**Keywords:** candidiasis, mammalian animal models, non-mammalian animal models

## Abstract

Candidiasis is a multifaceted fungal disease including mucosal-cutaneous, visceral, and disseminated infections caused by yeast species of the genus *Candida*. *Candida* infections are among the most common human mycoses. *Candida* species are the third to fourth most common isolates from bloodstream infections in neutropenic or immunocompromised hospitalized patients. The mucosal-cutaneous forms—particularly vaginal infections—have a high prevalence. Vaginitis caused by *Candida* species is the second most common vaginal infection. Hence, candidiasis is a major subject for research, including experimental in vivo models to study pathogenesis, prevention, or therapy of the disease. The following review article will focus on various experimental in vivo models in different laboratory animals, such as mammals (mice, rats, rabbits), the fruit fly–*Drosophila melanogaster*, the larvae of the moth *Galleria mellonella*, or the free-living nematode *Caenorhabditis elegans.* The review will describe the induction of the different clinical forms of candidiasis in the various models and the validity of such models in mimicking the human clinical situations. The use of such models for the assessment of antifungal drugs, evaluation of potential vaccines to protect before candidiasis, exploration of *Candida* virulence factors, and comparison of pathogenicity of different *Candida* species will be included in the review. All of the above will be reported as based on published studies of numerous investigators as well as on the research of the author and his group.

## 1. Introduction

Candidiasis is a multifaceted fungal disease including mucosal-cutaneous, visceral, and disseminated infections caused by yeast species of the genus *Candida*. *Candida* infections are among the most common human mycoses [1].

*Candida* species are the third to fourth most common isolates from bloodstream infections in neutropenic or immunocompromised hospitalized patients, primarily from intensive care units (ICUs) [2]. The Global Action Fund for Fungal Infections (Gaffi) [3] data indicate that ~300,000 cases of candidemia per year are predicted worldwide.

The mucosal-cutaneous forms—particularly vaginal infections—have a high prevalence. Vaginitis caused by *Candida* species is the second most common vaginal infection [4].

Gaffi data reveal that about 75 million women will suffer from recurrent vaginal infections. Oral thrush is an additional common mucosal *Candida* infection, afflicting over 9 million people [3].

Hence, candidiasis is a major subject for research, including experimental in vivo models to study pathogenesis, prevention, or therapy of the disease.

The following overview article aims to describe various experimental in vivo models in different laboratory animals, such as mammals (mice, rats, rabbits) [5], the fruit fly *Drosophila melanogaster* [6], the larvae of the moth *Galleria mellonella* [7], or the free-living nematode *Caenorhabditis elegans* [8]. The review will describe the induction of the different clinical forms of candidiasis in the various models and the validity of such models in mimicking the human clinical situations. The review will relate to models focusing on the evaluation of the efficacy of antifungal drugs in the treatment of the different clinical entities of candidiasis, or on evaluation of vaccines to assess protection before candidiasis. Exploration of *Candida* virulence factors or comparison of pathogenicity of different *Candida* species will be included in the review as well. All of the above will be reported based on published studies of numerous investigators, as well as on the research of the author and her group.

In order to mimic a human host, a mammalian animal would seem most rational; indeed: mice, rats, guinea-pigs and rabbits are the oldest known animal models. *Candida* experimental infections in these animals have been reported since the late 19th century [9].

## 2. The Mouse Model

Among the rodents, mice are the most widely-used animal models. A variety of clinical *Candida* entities have been studied in such models, including the mucosal oral or vaginal infections, the gastro-intestinal (GI), or deep-seated and systemic forms of candidiasis that have been induced experimentally in outbred and inbred mice strains. The most commonly used outbred mice strains are the ICR and Swiss white mice. Various inbred mice strains have been developed over the years for experimental work, of which the most commonly used strains in *Candida* studies are the BALB/C and BL 57 [10,11]. The author’s studies involved mostly the ICR strain for studies of systemic, vaginal, and oral *Candida* infections [12,13,14].

With the aim of mimicking the clinical situations in humans as closely as possible, experimental *Candida* infections can be induced not only in naïve mice, but also in mice rendered immunocompromised by pretreatment with various cytotoxic agents, such cyclophosphamide (CY) [12,15], 5-fluorouracyl (5FU) [16], or irradiation [16]. Other debilitating conditions can also be elicited in mice, such as experimental diabetes by pretreatment with streptozotocin [13] or continuous estrus stage by inoculation with estrogen [17].

Furthermore, the route of induction of infection may vary as well: systemic *Candida* infection can be induced by intravenous (IV) inoculation of the fungus, generally into the tail vein—a model widely used by most investigators [18,19], including the author [12,15]. Intraperitoneal (IP) injection is another route for the induction of systemic infection [20]. The GI route may also lead to a disseminated *Candida* infection [16].

The mouse model was intensively explored in studies aiming to elucidate mechanisms of pathogenicity and fungal virulence attributes [21], or comparison of virulence of different *Candida* species or strains within a specific species [15]. Lionakis et al. [18] studied organ-specific immune responses in experimental invasive candidiasis.

Great importance is given to studies exploring the activity of new antifungal drugs in the mouse model [19,22], both in naïve and compromised mice, which represents activity in vivo and is thus an essential step in drug development. Such studies may also focus on the antifungal activity of the drug in terms of its pharmacokinetic characteristics, such as tissue distribution, excretion, or other pharmacokinetic parameters [23].

The mouse model is also suitable for exploring of the immune responses elicited by the fungus and evaluating possible induction of immunity to the infection. This is an essential step in the assessment of the possibility for development of a vaccine. The immunization step is then followed by a challenge of the immunized animals with live microorganisms, and the degree of protection to withstand the challenge is assessed in comparison to non-vaccinated control animals [20]. Such experiments were extensively reported in the literature with various *Candida* immunogens, such as killed *Candida*, attenuated live organisms, or various subcellular *Candida* fractions [24,25,26,27], including studies by the author’s group using *Candida* ribosomes as an immunogen [20].

Preventive measures to protect before *Candida* infection may include antifungal coverage or vaccination. Another approach to prevention may be based on interference in the infectious process by inhibition of specific steps in the process. An example of this approach is the use of inhibition in the step of adherence to host tissues—an initial step of the infectious process in the evolution of candidiasis. Examples of such studies can be demonstrated by competitive binding of adhesins or receptors to inhibit the adhesion process [28].

### 2.1. Induction of Infection

#### 2.1.1. Systemic Infection

In the author’s group, most studies involved 4–6 week-old female ICR mice which were rendered immunocompromised by IP inoculation of 200 mg/kg cyclophosphamide (CY) [12,15]. Three days post-CY inoculation (peak of immunosuppression, as demonstrated by low numbers of white blood cells and a decrease in weight), the mice were inoculated IV into the tail vein with 10^4^ *Candida albicans* yeasts/mouse.

This mode of infection induction leads to systemic candidiasis suitable for the evaluation of antifungal drugs, immune responses, or comparison of the pathogenicity of *C. albicans* strains.

Infection was monitored for a period of up to 30 days and assessed by survival rate (%), mean survival time (MST), and fungal burden, as determined by enumeration of *Candida* colony forming units (CFU) in the kidneys [12,15]. In some experiments, evaluation also included histopathological examination of kidney tissues. In specific experiments, other organs, such as lungs, liver, spleen, or brain were also examined [23].

Naïve mice were inoculated with a higher inoculum: 5 × 10^4^ organisms/mouse.

**Figure 1:**
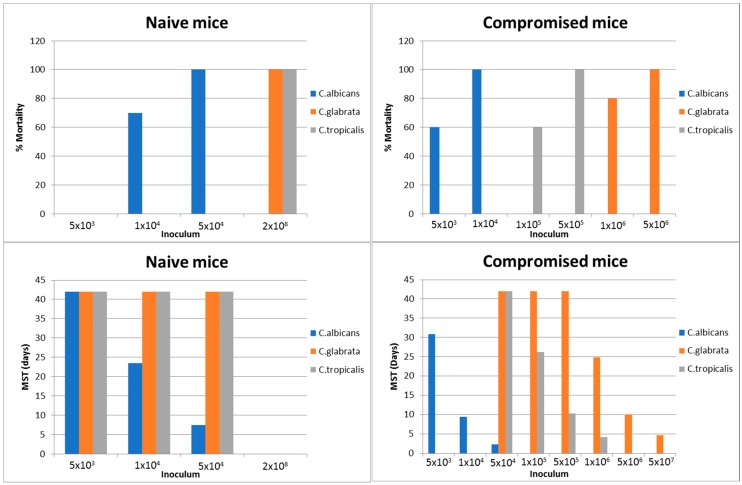
Systemic candidiasis in naïve and compromised ICR mice. Adapted from Schadkchan and Segal [29,30]; Naïve and compromised mice were injected intravenously; Follow-up of 42 days; Data of % survival refer to inoculum which resulted in 100% mortality; MST = Mean survival time.

Infection by non-albicans *Candida* species (*C. tropicalis*, *C. glabrata*, *C. krusei*, etc.) required much higher inoculum to elicit infection (see Figure 1).

Intraperitoneal (IP) inoculation of *C. albicans* can also lead to systemic candidiasis. However, in this model, higher fungal inoculum is required [20].

#### 2.1.2. Gastrointestinal (GI) Infection

*Candida albicans* is a human commensal and part of the human gastrointestinal (GI) mycobiota, from where it can spread under specific conditions into other sites of the body and cause a variety of clinical entities, including systemic candidiasis [1]. Although *C. albicans* is not part of the mouse mycobiota (they harbor *Candida pintolopesii*), mice have been used for experimental GI candidiasis.

We investigated the interaction of *C. albicans* with the GI tissue in ICR mice treated with cytotoxic anti-cancer drugs methotrexate (MTX) or 5-fluorouracil (5FU) to mimic human situations [16].

We adapted an experimental model of fatal systemic candidiasis originating from the gastrointestinal (GI) tract of compromised mice [16]. Female ICR mice were compromised by a single anti-cancer treatment: irradiation (4 or 6 Greys i.e., 400–600 rads), methotrexate (MTX) (3 mg per mouse, intraperitoneally), or 5-fluorouracil (5FU) (200 mg kg^−1^, intravenously). Three days later, compromised and control mice were administered orally with *C. albicans*. Morbidity and mortality due to candidiasis were monitored for 30 days post-Candidal inoculation. Increased and longer GI colonization was noted among the MTX and 5FU treated or irradiated mice. The stomach was found to be the major part of the GI tract involved in fungal colonization. It is worth emphasizing that a significant number (53.8–83.3%) of the anti-cancer-treated mice developed systemic candidiasis originating from the GI tract, which was fatal in 30–80% of the infected animals. *Candida* could be found in the liver, spleen, and kidneys. In systemically-infected animals, Candidal antigen was demonstrated in the serum, and fungal abscesses containing *C. albicans* were observed in the liver, kidneys, and spleen.

#### 2.1.3. Vaginal Infection

Experimental *Candida* vaginitis can be induced in mice and rats.

Very early observations [31] indicated that the optimal timing for the induction of vaginal infection is during the estrus stage of the mice. The estrus stage is characterized by the massive appearance of epithelial cells in the vaginal exudate. The estrus-cycle of mice is 3–4 days in duration. Mice infected during the estrus stage will develop the infection, which may disappear spontaneously afterward. Hence, most investigators—including the author—use models in which a constant estrus state is maintained by inoculating female mice with estradiol benzoate [17] 3–4 days prior to inoculation with *Candida*. Infection is induced by intravaginal inoculation of 10^7^ *C. albicans* yeast cells, and can be maintained by repeated weekly inoculations of estradiol benzoate.

Diabetic women suffer from higher rates of *Candida* vaginitis [32]. In the frame of studies to inhibit adhesion of *Candida* to host tissues and thereby prevent infection, we explored this aspect in an experimental infection in diabetic mice. Mice were rendered diabetic by intraperitoneal (IP) injection of 160 mg/kg streptozotocin [13]. Diabetic state was apparent 2–7 days later. Mice were inoculated intra-vaginally with 10^7^–10^10^ *C. albicans* and followed-up for 35 days. The diabetic mice developed a massive infection of long duration while in the non-diabetic control mice the infection was less massive and cleared earlier (see Table 1). Furthermore, the fungal burden in the vaginal wash—as measured by *Candida* CFU—was 10-fold lower in the non-diabetic mice than in the diabetic counterparts. Thus, this model mimics a real clinical situation and therefore seems to be valid.

## 3. The *Drosophila melanogaster* Model

Non-mammalian animal models are of significance for biological and medical research as they are easier and simpler to handle, less costly, and save manpower. This rationale led to attempts seeking non-mammalian alternatives. Among several non-mammalian animal models in-use in biological and medical research, the oldest is *Drosophila melanogaster*.

*Drosophila melanogaster* is a small (~3 mm long) fruit fly and it has served as model—particularly in genetics and developmental biology—for almost a century. It is a small animal with a short life cycle of just two weeks, and is cheap and easy to keep large numbers. Mutant flies with defects in any of several thousand genes are available, and the entire genome has recently been sequenced [33].

Its importance and vast use in biological research was emphasized by the Nobel prize in physiology in 1995 given to Ed Lewis, Christiane Nusslein-Volhard, and Eric Wieschaus.

Chamilos et al. [6] developed a model in *Drosophila melanogaster* and studied fungal virulence attributes and drug discovery. Specifically, these investigators developed a model of candidiasis in *Toll* (*Tl*)–deficient *Drosophila melanogaster*. *C. parapsilosis* was less virulent than *C. albicans* in the *Tl* mutant flies, mimicking the human condition. Comparison of the findings of the attenuated *cph1*/*cph1* and *efg1*/*efg1 C. albicans* mutant in the mouse model with those in the T1 mutant flies indicated similarity. Hence, these researchers concluded that the *Drosophila melanogaster* model is a promising model for large-scale studies of virulence mechanisms and antifungal drug activity in candidiasis. Glitenberg et al. [33] assessed the virulence of various clinical isolates of *C. albicans* in wild-type *Drosophila melanogaster*, and found that the virulence of the isolates correlated with that noted previously in the murine model. Hence, these investigators also concluded that the *Drosophila* model is a relevant model to study *Candida* infection.

A different group of investigators [34] used immune-deficient *Drosophila melanogaster* to explore the innate immune response to human fungal pathogens. They showed that specific *C. albicans* mutants differing in virulence in murine model exhibited a similar pattern of virulence in the *Drosophila* model. Moreover, in this model they could detect virulence characteristics not detected in an immunocompetent model.

It is of interest that in *Drosophila*, *C. albicans* can change its morphology from yeast to the pseudohyphal form, similar to the situation occurring during infection in mammalian hosts [35]. Furthermore, a very recent publication [36] asks an intriguing question—whether the fruit fly could be a potential vector of opportunistic pathogens, since they isolated 18 species of fungi, including *Candida* and *Aspergillus* species, from wild fruit flies.

## 4. The *Caenorhabditis elegans* Model

*Caenorhabditis elegans* is a small (about 1 mm in length) free-living transparent nematode found in temperate soil environments, which has been used as an animal model in developmental biology research since 1974 [8,37].

The advantages of this model system lie in its being a eukaryotic multicellular organism and its being transparent, enabling easy observation. In addition, *C. elegans* is cheap and easy to grow, can be frozen without losing viability, and hence can be stored easily for long periods of time. *C. elegans* does not poses an adaptive immune system but only an innate immune system by which to defend itself [38].

In relevance to fungi, *C. elegans* has been involved, among others, in the areas of antimicrobial drug discovery studies [39]; studies related to antifungal immune defenses [40] and antifungal efficacy against non-*C. albicans* species [41].

Pukkila-Worle et al. [40] showed that live *C. albicans* can establish an intestinal infection in *C. elegans*, while heat-killed organisms cannot. These authors also reported that by transcription profiling of *C. elegans* they demonstrated that exposure to *C. albicans* stimulated a host response in this nematode. The response is mediated through “pattern recognition”, recognizing pathogen-associated molecular patterns (PAMPs).

Of interest is the statement of Ewbank and Zagusti [39] that *C. elegans* cannot completely replace mammalian systems due to various differences, among which a major one is the inability of *C. elegans* to grow at the mammalian body temperature of 37 °C.

Scorzoni et al. [41] studied antifungal efficacy against *C. krusei* infection in two non-mammalian in vivo systems: *C. elegans* and *Galleria mellonella*. The authors found a correlation between the in vivo data and the in vitro susceptibility assays’ data, which lends validity to the in vivo models.

## 5. The *Galleria mellonella* Model

Another non-mammalian animal model introduced into biological-medical research in the late 1990s–early 2000s which is currently used by many investigators is the larvae of the moth *Galleria mellonella* [7]. In this case as well, the rationale is to save human resources and costs associated with use of mammalian animal models. The larvae are commercially available and easy to handle. Furthermore, due to the strong structural and functional similarities between the immune response of insects and the innate immune responses of mammals [42], insects can be used to study alterations in microbial virulence.

*Galleria mellonella* has been used as a model for the in vivo assessment of the pathogenicity of bacterial and fungal species [43,44,45,46].

The author’s group studied the comparative pathogenicity of *C. albicans* isolates from blood-stream *Candida* infection vs. isolates from vaginitis, both in *Galleria mellonella* and in a mouse model [15].

Table 2 shows the results of such tests. It can be noted that in the control strain CBS 562 and the blood isolate S14, the data of both models showed comparable results, with both strains showing the highest virulence. However, in other strains (M33 and M39), differences between the two models were noted. It should also be added that while both models enabled the assessment of mortality rate as a criterion of pathogenicity, the mouse model also enabled the assessment of morbidity, as determined by quantitative evaluation of mouse kidney colonization.

It is of interest that the literature demonstrates differences between researchers. While Amorim-Vaz et al. [46] observed a discrepancy in the results obtained in the two models, Hirakawa et al. [47], Brenan et al. [48], and Slater et al. [45] reported similar results in both models. Thus, in any in vivo assessment, research differences between models among researchers should be taken into consideration.

The *Galleria* model has also been used for testing antimicrobial efficacy [49] and the assessment of the pharmacokinetic characteristics of antimicrobial agents [50]. Mesa-Aranga et al. [51] tested the efficacy of antifungal drugs against *Candida tropicalis*, and Ames et al. [49] evaluated the activity of antifungals against *Candida glabrata.* Furthermore, the group of Astvad et al. [50] studied the pharmacokinetics of fluconazole in the larvae of *Galleria mellonella* in comparison to liquid chromatography.

Although some studies [52] indicated that transfer of the *Galleria mellonella* hemolymph may transfer immunity to *Pseudomonas aeruginosa*, no studies regarding anti-*Candida* or anti other-fungal vaccine involving vaccination and challenge with live *Candida* organisms has been described. Thus, *Galleria mellonella* is a system in which many of the biological aspects of microbial organisms can be investigated, however not to the same extent as in mammalian systems.

## 6. Summary

In summary, this article focused on experimental *Candida* infections in four different models: the mouse model as a representative of a mammalian model and three non-mammalian models: *Drosophila melanogaster*, *Caenorhabditis elegans*, and *Galleria mellonella*. All three non-mammalian models were generally compared to the traditional mammalian model. Many of the data correlated well with the mammalian model, but some were not compatible, suggesting that caution should be used in analysis and interpretation.

The major advantages of the non-mammalian models lie in the economy of human resources, affordable costs, and ease of handling. It should, however, be added in this context, that while the mammalian model enables research regarding pathogenicity, drug activity, and drug pharmacokinetics, as well as vaccination attempts involving immunization and challenge with the microbe to assess the elicited protection and immune responses, the non-mammalian models are not suitable for classical microbial vaccination and challenge studies, nor for organ colonization assessments. In addition, since some of the models (e.g., *C. elegans*) are not functional at the mammalian body temperature (37 °C) and lack an adaptive immune system, the validity of such models in reference to the human host is diminished.

Thus, the authors ultimately believe that the mammalian model more closely represents the human situation.

## Figures and Tables

**Table 1:** Vaginal infection induced in diabetic and naïve mice.

Days After Inoculation	Diabetic Mice with Estrus (% Infected)	Diabetic Mice Without Estrus (% Infected)	Naïve Mice
1	100	92	70
3	100	92	20
5	83	58	10
7	83	55	0
14	67	55	0
21	50	55	0
28	50	55	0
35	33	55	0

Adapted from Segal and Yosef-Lev [13].

**Table 2:** Comparison of pathogenicity of *Candida albicans* strains in mouse and *Galleria mellonella*.

	Mouse Model		*Galleria* Model	
Strain	MST (Days)	SD	MST (Days)	SD
S2	28.27	4.9	2.75	1.95
S11	27.87	4.7	2.00	0.97
S19	27.67	4.0	1.90	0.80
S5	27.5	5.9	2.18	1.20
S14	12.6	9.3	1.70	0.75
CBS	4.47	2.2	1.31	0.51
M33	16.47	6.7	3.33	2.08
M32	17.93	9.5	2.65	1.37
M42	21.67	9.4	1.40	1.35
M29	24.64	8.5	2.95	1.84
M39	27.53	5.5	1.40	0.78
M26	29.53	1.8	2.45	1.25

Adapted from Frenkel et al. [15]; Mice were inoculated with bloodstream (S) and vaginal (M) *C. albicans* strains and mean survival time was determined (MST); No significant difference was observed between the M and S strains in both models.

## References

[1] Edwards J.E., Bennett J.E., Dolin R., Blaser M.J. (2015). *Candida* species. Principles and Practice of Infectious Diseases.

[2] Pfaller M.A., Diekema D.J. (2007). Epidemiology of invasive candidiasis: A persistent public health problem. Clin. Microbiol. Rev..

[3] Global Action Fund for Fungal Infections. https://www.gaffi.org..

[4] Sobel J.D. (2007). Vulvovaginal candidosis. Lancet.

[5] Romani L. (1999). Animal Models for Candidiasis. Current Protocols in Immunology.

[6] Chamilos G., Lionakis M.S., Lewis R.E., Lopez-Ribot J.L., Saville S.P., Albert N.D., Halder G., Kontoyiannis D.P. (2006). *Drosophila* melanogaster as a facile model for large-scale studies of virulence mechanisms and antifungal drug efficacy in *Candida* species. J. Infect. Dis..

[7] Fuchs B.B., O’Brien E., El Khoury J.B., Mylonakis E. (2010). Methods for using *Galleria mellonella* as a model host to study fungal pathogenesis. Virulence.

[8] Brenner S. (2009). In the Beginning Was the Worm. Genetics.

[9] Joly V., Yeni P., Zak O., Sande M. (1999). Rodent models of *Candida* sepsis. Handbook of Animal Models of Infection.

[10] Inbred Strains of Mice: BALB. http://www.informatics.jax.org/inbred_strains/mouse/docs/BALB.shtml.

[11] Aurora’s Guide to Mouse Colony Management at MIT. https://ki.mit.edu/files/ki/cfile/sbc/escell/mouseManagement.pdf.

[12] Semis R., Mendlovic S., Polacheck I., Segal E. (2011). Activity of an Intralipid formulation of nystatin in murine systemic candidiasis. Int. J. Antimicrob. Agents.

[13] Segal E., Josef-Lev A. (1995). Induction of Candidal vaginitis in diabetic mice and attempts to prevent the infection. J. Med. Vet. Mycol..

[14] Segal E., Baranetz T., Sandovsky-Losica H., Gov Y., Teicher S., Dayan D. (1999). Experimental oral murine candidiasis and attempts of prevention. J. Med. Mycol..

[15] Frenkel M., Mandelltat M., Alastruey-Izquierdo A., Mendlovic S., Semis R., Segal E. (2016). Pathogenicity of *Candida albicans* isolates from bloodstream and mucosal candidiasis assessed in mice and *Galleria mellonella*. J. Mycol. Med..

[16] Sandovsky losica H., Barrnea L., Segal E. (1992). Fatal Systemic Candidiasis of Gastrointestinal Origin-an Experimental-Model in Mice Compromised by Anticancer Treatment. J. Med. Vet. Mycol..

[17] Segal E., Gottfried L., Lehrer N. (1988). Candidal Vaginitis in Hormone-Treated Mice—Prevention by a Chitin Extract. Mycopathologia.

[18] Lionakis M.S., Lim J.K., Lee C.C., Murphy P.M. (2011). Organ-specific innate immune responses in a mouse model of invasive candidiasis. J. Innate Immunity.

[19] Mohamed H.A., Radwan R.R., Raafat A.I., Ali A.E. (2018). Antifungal activity of oral (Tragacanth/acrylic acid) Amphotericin B carrier for systemic candidiasis: In vitro and in vivo study. Drug Deliv. Transl. Res..

[20] Levy R., Segal E., Eylan E. (1981). Protective Immunity against Murine Candidiasis Elicited by *Candida-albicans* Ribosomal Fractions. Infect. Immunity.

[21] Schulz B., Weber K., Schmidt A., Borg-von Zepelin M., Ruhnke M. (2011). Difference in virulence between fluconazole-susceptible and fluconazole-resistant *Candida albicans* in a mouse model. Mycoses.

[22] Sanchis M., Capilla J., Castanheira M., Martin-Vicente A., Sutton D.A., Fothergill A.W., Wiederhold N.P., Guarro J. (2016). Voriconazole minimum inhibitory concentrations are predictive of treatment outcome in experimental murine infections by *Candida glabrata*. Int. J. Antimicrob. Agents.

[23] Semis R., Nili S.S., Munitz A., Zaslavsky Z., Polacheck I., Segal E. (2012). Pharmacokinetics, tissue distribution and immunomodulatory effect of intralipid formulation of nystatin in mice. J. Antimicrob. Chemother..

[24] Segal E., Elad D. (2006). Fungal vaccines and immunotherapy. J. Mycol. Med..

[25] Segal E. (2017). Testing Antifungal Vaccines in an Animal Model of Invasive Candidiasis and in Human Mucosal Candidiasis. Methods Mol. Biol..

[26] Ahmad E., Zia Q., Fatima M.T., Owais M., Saleemuddin M. (2015). Vaccine potential of plasma bead-based dual antigen delivery system against experimental murine candidiasis. Int. J. Biol. Macromol..

[27] Saville S.P., Lazzell A.L., Chaturvedi A.K., Monteagudo C., Lopez-Ribot J.L. (2009). Efficacy of a Genetically Engineered *Candida albicans tet-NRG1* Strain as an Experimental Live Attenuated Vaccine against Hematogenously Disseminated Candidiasis. Clin. Vaccine Immunol..

[28] Segal E., Kahane I., Ofek I. (1996). Inhibition of *Candida* adhesion to prevent candidiasis. Toward Anti Adhesion Therapy for Microbial Diseases of Advances in Experimental Medicine and Biology.

[29] Shadkchan Y., Segal E. (1999). Antifungal activity of amphotericin B-lipid admixtures in experimental systemic candidosis in naive mice. J. Antimicrob. Chemother..

[30] Shadkchan Y., Segal E. (2001). Treatment of experimental candidosis with amphotericin B-Intralipid admixtures in immunocompromised mice. J. Antimicrob. Chemother..

[31] Taschdjian C.L., Reiss F., Kozin P.J. (1960). Experimental vaginal candidiasis in mice; its implications for superficial candidiasis in humans. J. Investig. Dermatol..

[32] Peer A.K., Hoosen A.A., Seedat M.A., van den Ende J., Omar M.A. (1993). Vaginal Yeast Infections in Diabetic Women. S. Afr. Med. J..

[33] Glittenberg M.T., Silas S., MacCallum D.M., Gow N.A., Ligoxygakis P. (2011). Wild-type *Drosophila melanogaster* as an alternative model system for investigating the pathogenicity of *Candida albicans*. Dis. Model. Mech..

[34] Alarco A.M., Marcil A., Chen J., Suter B., Thomas D., Whiteway M. (2004). Immune-deficient *Drosophila melanogaster*: A model for the innate immune response to human fungal pathogens. J. Immunol..

[35] San-Blas G., Travassos L.R., Fries B.C., Goldman D.L., Casadevall A., Carmona A.K., Barros T.F., Puccia R., Hostetter M.K., Shanks S.G. (2000). Fungal morphogenesis and virulence. Med. Mycol..

[36] Ramirez-Camejo L.A., Maldonado-Morales G., Bayman P. (2017). Differential Microbial Diversity in *Drosophila melanogaster*: Are Fruit Flies Potential Vectors of Opportunistic Pathogens?. Int. J. Microbiol..

[37] Sulston J.E., Brenner S. (1974). The DNA of *Caenorhabditis elegans*. Genetics.

[38] Ewbank J.J., Pujol N. (2016). Local and long-range activation of innate immunity by infection and damage in *C. elegans*. Curr. Opin. Immunol..

[39] Ewbank J.J., Zugasti O. (2011). *C. elegans*: Model host and tool for antimicrobial drug discovery. Dis. Model. Mech..

[40] Pukkila-Worley R., Feinbaum R.L., McEwan D.L., Conery A.L., Ausubel F.M. (2014). The evolutionarily conserved mediator subunit MDT-15/MED15 links protective innate immune responses and xenobiotic detoxification. PLoS Pathog..

[41] Scorzoni L., de Lucas M.P., Mesa-Arango A.C., Fusco-Almeida A.M., Lozano E., Cuenca-Estrella M., Mendes-Giannini M.J., Zaragoza O. (2013). Antifungal efficacy during *Candida krusei* infection in non-conventional models correlates with the yeast in vitro susceptibility profile. PLoS ONE.

[42] Moghaddam M.R.B., Tonk M., Schreiber C., Salzig D., Czermak P., Vilcinskas A., Rahnamaeian M. (2016). The potential of the *Galleria mellonella* innate immune system is maximized by the co-presentation of diverse antimicrobial peptides. Biol. Chem..

[43] Ramarao N., Nielsen-Leroux C., Lereclus D. (2012). The Insect *Galleria mellonella* as a Powerful Infection Model to Investigate Bacterial Pathogenesis. J. Vis. Exp..

[44] Coleman J.J., Muhammed M., Kasperkovitz P.V., Vyas J.M., Mylonakis E. (2011). Fusarium pathogenesis investigated using *Galleria mellonella* as a heterologous host. Fungal Biol..

[45] Slater J.L., Gregson L., Denning D.W., Warn P.A. (2011). Pathogenicity of *Aspergillus fumigatus* mutants assessed in *Galleria mellonella* matches that in mice. Med. Mycol..

[46] Amorim-Vaz S., Delarze E., Ischer F., Sanglard D., Coste A.T. (2015). Examining the virulence of *Candida albicans* transcription factor mutants using *Galleria mellonella* and mouse infection models. Front. Microbiol..

[47] Hirakawa M.P., Martinez D.A., Sakthikumar S., Anderson M.Z., Berlin A., Gujja S., Zeng Q.D., Zisson E., Wang J.M., Greenberg J.M. (2015). Genetic and phenotypic intra-species variation in *Candida albicans*. Genome Res..

[48] Brennan M., Thomas D.Y., Whiteway M., Kavanagh K. (2002). Correlation between virulence of *Candida albicans* mutants in mice and *Galleria mellonella* larvae. FEMS Immunol. Med. Microbiol..

[49] Ames L., Duxbury S., Pawlowska B., Ho H.L., Haynes K., Bates S. (2017). *Galleria mellonella* as a host model to study *Candida glabrata* virulence and antifungal efficacy. Virulence.

[50] Astvad K.M.T., Meletiadis J., Whalley S., Arendrup M.C. (2017). Fluconazole Pharmacokinetics in *Galleria mellonella* Larvae and Performance Evaluation of a Bioassay Compared to Liquid Chromatography-Tandem Mass Spectrometry for Hemolymph Specimens. Antimicrob. Agents Chemother..

[51] Mesa-Arango A.C., Forastiero A., Bernal-Martinez L., Cuenca-Estrella M., Mellado E., Zaragoza O. (2013). The non-mammalian host *Galleria mellonella* can be used to study the virulence of the fungal pathogen *Candida tropicalis* and the efficacy of antifungal drugs during infection by this pathogenic yeast. Med. Mycol..

[52] De Verno P.J., Aston W.P., Chadwick J.S. (1983). Transfer of Immunity against *Pseudomonas-aeruginosa* P11-1 in *Galleria-mellonella* Larvae. Dev. Comp. Immunol..

